# Induction of cancer neoantigens facilitates development of clinically relevant models for the study of pancreatic cancer immunobiology

**DOI:** 10.1007/s00262-023-03463-x

**Published:** 2023-05-13

**Authors:** Usman Y. Panni, Michael Y. Chen, Felicia Zhang, Darren R. Cullinan, Lijin Li, C. Alston James, Xiuli Zhang, S. Rogers, A. Alarcon, John M. Baer, Daoxiang Zhang, Feng Gao, Christopher A. Miller, Qingqing Gong, Kian-Huat Lim, David G. DeNardo, S. Peter Goedegebuure, William E. Gillanders, William G. Hawkins

**Affiliations:** 1 Department of Surgery, Washington University School of Medicine, Campus Box 8109, 660 S. Euclid Ave., St. Louis, MO 63110 USA; 2 Department of Medicine, Washington University School of Medicine, St. Louis, MO USA; 3 McDonnell Genome Institute, Washington University School of Medicine, St. Louis, MO USA; 4 Department of Pathology and Immunology, Washington University School of Medicine, St. Louis, MO USA; 5 Alvin J. Siteman Cancer Center at Barnes-Jewish Hospital and Washington University School of Medicine, St. Louis, MO USA

**Keywords:** Pancreatic cancer, Tumor immunology, Cancer neoantigen, Immune checkpoint inhibition

## Abstract

Neoantigen burden and CD8 T cell infiltrate are associated with clinical outcome in pancreatic ductal adenocarcinoma (PDAC). A shortcoming of many genetic models of PDAC is the lack of neoantigen burden and limited T cell infiltrate. The goal of the present study was to develop clinically relevant models of PDAC by inducing cancer neoantigens in KP2, a cell line derived from the KPC model of PDAC. KP2 was treated with oxaliplatin and olaparib (OXPARPi), and a resistant cell line was subsequently cloned to generate multiple genetically distinct cell lines (KP2-OXPARPi clones). Clones A and E are sensitive to immune checkpoint inhibition (ICI), exhibit relatively high T cell infiltration, and have significant upregulation of genes involved in antigen presentation, T cell differentiation, and chemokine signaling pathways. Clone B is resistant to ICI and is similar to the parental KP2 cell line in terms of relatively low T cell infiltration and no upregulation of genes involved in the pathways noted above. Tumor/normal exome sequencing and in silico neoantigen prediction confirms successful generation of cancer neoantigens in the KP2-OXPARPi clones and the relative lack of cancer neoantigens in the parental KP2 cell line. Neoantigen vaccine experiments demonstrate that a subset of candidate neoantigens are immunogenic and neoantigen synthetic long peptide vaccines can restrain Clone E tumor growth. Compared to existing models, the KP2-OXPARPi clones better capture the diverse immunobiology of human PDAC and may serve as models for future investigations in cancer immunotherapies and strategies targeting cancer neoantigens in PDAC.

**Supplementary Information:**

The online version contains supplementary material available at 10.1007/s00262-023-03463-x.

## Background

Cancer neoantigens are antigens associated with genetic alterations present in tumors. These genetic alterations include nonsynonymous point mutations, insertion-deletion, and frameshift mutations resulting in altered amino acid sequences that can be recognized by the immune system [1, 2]. To be recognized by host immune cells as foreign antigens, neoantigens are expressed on the cell surface by major histocompatibility complex (MHC) molecules [3–5]. Due to their tumor-specificity, the response to neoantigens is less likely to be limited by central immune tolerance. It is hypothesized that cancer neoantigens play a critical role in priming and activating T cells against tumor cells [4, 6]. Furthermore, cancer neoantigens are an attractive target for personalized immunotherapies, including neoantigen vaccine therapy [2, 7–16].

Cancer neoantigens occur more frequently in tumors that harbor a high rate of somatic mutations, such as melanoma, non-small cell lung cancer, and microsatellite instability-high colorectal cancer [17–19]. Recent studies have demonstrated a significant correlation between the efficacy of checkpoint immunotherapy and tumor mutational burden. Yarchoan et al. and others have shown the number of amino acid-coding somatic mutations in the genome is strongly associated with the activity of immune checkpoint inhibition (anti-PD1 and anti-CTLA4) across multiple cancers [20–24]. These observations suggest an important role for cancer neoantigens and overall mutational burden as a potential biomarker for response to immunotherapy.

Pancreatic ductal adenocarcinoma (PDAC) has been largely refractory to immunotherapy, including immune checkpoint inhibition. In addition to a densely fibrotic stroma and a prominent myeloid cell infiltration that contribute to the therapeutic resistance, the failure of immunotherapy in PDAC has also been attributed to a lower average mutational burden (2.64 somatic mutations per megabase of coding sequence) compared to immunotherapy-sensitive tumors such as melanoma (which harbor 13.5 mutations per Mb) [19, 25–31]. Bailey et al. have shown that while PDAC tumors express a moderate number of targetable cancer neoantigens (mean number of coding mutations = 62), an effective immune response cannot be generated due to a predominantly immunosuppressive microenvironment [29, 32–35]. In other studies, the expression of high-quality neoantigens has been shown to contribute to PDAC immunogenicity and, in combination with abundant CD8 T cell infiltration, is associated with long-term survival benefit in patients [36].

KPC is a genetic model of PDAC that recapitulates human disease due to its activating mutations in *Kras (G12D)* and loss of *Trp53*. It is characterized by a dense desmoplastic stroma and an abundant myeloid cell infiltrate that promote an immunosuppressive microenvironment [37–41]. KPC tumors do not respond to anti-PD-1 and anti-CTLA4 therapy. These features make KPC an attractive preclinical model to study human disease. However, KPC tumors express few, if any, neoantigens derived from somatic mutations [38, 41]. To overcome the relative lack of cancer neoantigens in KPC and other models of PDAC, studies have utilized the expression of xenoantigens such as ovalbumin or murine mesothelin to successfully study the impact of tumor antigenicity on the tumor stroma and T cell-mediated immune response in the KPC model [38, 41, 42]. While expressing a single, strong antigen is useful in understanding the immune-modulatory mechanisms that drive neoantigen-specific immunity, it does not recapitulate the neoantigen burden in human PDAC. Therefore, there is an ongoing need for a PDAC mouse model that has a neoantigen burden similar to the human disease.

To develop a preclinical model that can be used to study PDAC immunobiology, we deployed a novel strategy of treating KP2 (a KPC-derived tumor line) with OXPARPi treatment, consisting of a mutagenic chemotherapy (oxaliplatin) and a small molecule PARP inhibitor (olaparib), to induce genetic alterations. We hypothesized that these genetic alterations would translate into an increased neoantigen burden. We then utilized pVAC-Seq suite of software tools [43] to identify candidate neoantigens in clones of OXPARPi treated KP2 (KP2-OXPARPi clones), and assessed their immunogenicity and ability to elicit antitumor immunity. To evaluate the translational significance of our findings, we analyzed human PDAC tissue to identify cancer neoantigens and performed in vitro assays to assess neoantigen-specific immune responses.

## Methods

### Study design

Washington University School of Medicine Institutional Animal Studies Committee approved all animal studies (Protocol Number 20190111). All preclinical studies were designed with the help of the Siteman Cancer Center Biostatistics Core. The core staff provided estimates for sample size, power calculations and help with data analysis. All experiments were replicated two to three times, and all critical observations were made with different cell lines. To blind the research team, only animal number, not treatment groups, was used when investigators made measurements or conducted analysis, e.g., Tumor weight and volume measurements, survival, flow cytometry analysis, and ELISPOT studies.

All human subjects research was reviewed and approved by the Washington University Institutional Review Board (IRB). The patient specimens were de-identified and were designated a 3-digit identification number, i.e., 8117–665 (Subject A), 8117–666 (Subject B), and 8117–667 (Subject C). Data from clinical trial NCT03122106 were obtained following informed consent, FDA, and IRB approval.

### Development of KP2-OXPARPi cell lines

Mice were maintained in the Washington University Division of Comparative Medicine Animal Care barrier facility. The KP2 cell line was derived from a tumor of a 6-month-old p48-CRE^+^/LSL-Lox Kras^G12D^/p53^flox/+^ mouse. KP2-OXPARPi cell lines were developed by treating KP2 cells, plated in a 6-well flat bottom plate, with 5% DMSO (Control), Oxaliplatin 10uM, Olaparib 5uM, and oxaliplatin 10uM and Olaparib 5uM in combination for a duration of 4 months. All tumor lines were maintained in DMEM-F12 (Gibco), supplemented by 10% FBS, and 1% penicillin/streptomycin (Gibco). Upon completion of treatment, individual KP2-OXPARPi clones (A through F) were separated using the limiting dilution technique as described (https://www.corning.com/catalog/cls/documents/protocols/Single_cell_cloning_protocol.pdf). All cell lines tested negative for mycobacterium avium subspecies paratuberculosis (MAP) and mycoplasma. To establish subcutaneous tumors, either 250,000 or 500,000 KP2 or KP2-OXPARPi clones were injected in 100ul of Matrigel (Corning) into the right flank of 8- to 12-week-old C57BL/6 NCr mice (Charles River). All animals were randomized and assigned treatment groups on Day 5 post-implant.

### In vivo inhibitors and neutralizing antibodies

Immunotherapy IgGs were given intraperitoneally; anti-PD-1 (200 μg per dose, clone RMP1–14) was given every 3 days, whereas anti-CTLA4 (250 μg per dose, clone UC10-4F10-11) were given every 4 days. Anti-CTLA4 was discontinued after four doses, whereas anti-PD-1 was continued until day 30. All were purchased from BioXCell. For T cell depletion, CD4- and CD8-neutralizing IgG antibodies (anti-mCD4 clone GK1.5 and anti-mCD8 clone 2.43, BioXCell) were administered via intraperitoneal injection every 4 days, with the first injection containing 500 μg before tumor implantation and subsequent injections containing 250 μg.

### Mouse tissue isolation and flow cytometry

Mice were euthanized by carbon dioxide asphyxiation. Tumor tissues were manually minced and digested in 25 ml of Dulbecco’s modified Eagle medium containing collagenase A (2 mg/ml) (Roche) and deoxyribonuclease I (DNase I) (Sigma-Aldrich) for 30 min at 37 °C. Digestion was quenched in 5 ml of FBS and filtered through a 40-μm filter. Single-cell suspensions were subsequently labeled with fluorophore-conjugated anti-mouse antibodies at recommended dilutions following the manufacturers’ recommendations. Data were acquired on X-20 (BD Biosciences) and analyzed using FlowJo software (Tree Star) (Supplementary Fig. 6).

### Immunohistochemistry (IHC)

Tissues were fixed in 10% formalin overnight (Fisher Scientific), incubated in graded ethanol, embedded in paraffin, and cut into 5-μm-thick sections. These Sects. (5-μm-thick) were air-dried and fixed in 4% paraformaldehyde (PFA) (Ted Pella Inc.). For CD8 + and CD4 + T cell analysis, tissues were stained using Bond RXm autostainer (Leica Biosystems). For all quantifications, whole tissue slide scans were obtained at 10 × magnification on Zeiss Axio Scan Z1 Brightfield/Fluorescence Slide Scanner. Whole tissue slide scans at 10 × magnification were analyzed with HALO software (Indica Labs).

### Human peripheral blood and tumor samples

Following informed consent, tissue and peripheral blood were collected from three consecutive patients undergoing surgical resection for pancreatic ductal adenocarcinoma (PDAC). Subjects A and B underwent a pancreaticoduodenectomy, and Subject C had a total pancreatectomy. At the time of collection, Subjects A and C had received no cancer-related treatment, while Subject B received neoadjuvant chemotherapy with an oxaliplatin-based regimen before undergoing surgery. Primary PDAC tissues were collected during surgical resection and verified by standard pathology. Leukapheresis was performed at Barnes Jewish Hospital. Blood was collected in vacuum tubes containing heparin or ethylenediaminetetraacetic acid (EDTA). Peripheral blood mononuclear cells (PBMC) were isolated through density centrifugation using Ficoll-Paque PLUS and cryopreserved as fetal bovine serum (FBS) with 10% dimethyl sulfoxide (DMSO).

### Nucleic acid isolation

**Murine tumors** DNA and RNA were isolated from fresh subcutaneous tumors according to previously published protocols from our institution [44].

**Human PDAC tissue** A board-certified pathologist reviewed the surgical pathology specimens and determined that the biospecimens from all three subjects had sufficient tumor cellularity to proceed with nucleic acid isolation. Five to six 1.5 mm punches were taken from formalin-fixed paraffin-embedded (FFPE) blocks from areas of estimated tumor cellularity of > 40%, using an H&E-stained slide as a guide. For nucleic acid isolation, RNA and DNA were purified from the punches using the Qiagen AllPrep FFPE Kit (catalog # 80,234, Germantown, MD, USA) following manufacturer’s instructions except for increased Proteinase K digestion length. As RNA degradation was a specific concern, we analyzed RNA integrity numbers (RIN) and fragment distribution values, DV200, which reflects the percentage of RNA fragments > 200 base pairs.

### Identification of candidate neoantigens

**Murine studies** Total RNA sequencing libraries were created and sequenced on the Illumina NovaSeq 6000 platform. Paired-end sequence reads were aligned to the GRCm38 (mm10) reference genome using HISAT version 2.1.0 [45]. Read counts were extracted using bam-readcount [https://joss.theoj.org/papers/10.21105/joss.03722]. Expression matrices were generated using kallisto version 0.43.1 [46], against Ensembl mouse build 95, and normalized using the edgeR package for R (version 3.20.9) [47]. For the identification of immunogenic mutations in KP2-OXPARPi clones, sequence data were aligned to the mm10 reference genome, then variants called using GATK Haplotype Caller. Blocks of recurrent mutations caused by divergence from the mm10 reference sequence were filtered if 3 or more mutations occurred within 100 kb. Removed sites were reviewed to confirm minimal impact upon coding mutation burden. These variants were then annotated, and coding mutations were fed into pVACtools version 2.0. Mutations and flanking bases were translated into the corresponding amino-acid sequences, then evaluated using both H-2D^b^/H-2 Kb (MHCflurry, NetMHC, NetMHCcons, NetMHCpan, PickPocket, SMM, SMMPMBEC) and H-2IA^b^ peptide-binding algorithms (NNalign, SMMalign, NetMHCIIpan). Any mutation with a median IC50 value of < 1000 nm was considered a potential candidate neoantigen. The neoantigen sequences were 8-to 11 amino acids in length for H-2D^b^/H-2 Kb and 15 amino acids in length for H-2IA^b^ algorithms. The candidate neoantigens were ranked based on their binding affinity, degree of fold change between mutant and wild-type alleles, and DNA variant allele fraction (VAF).

**Human studies** Tumor/normal DNA exome sequencing and cDNA-capture sequencing of tumor RNA was performed to compile a list of expressed somatic mutations, using the pipeline detailed at https://github.com/genome/analysis-workflows/releases/tag/v1.5.0. Neoantigens were predicted using pVAC-Seq version 2.0, a computational tool for neoantigen identification and prioritization [43, 48]. Briefly, the expressed mutations and flanking bases were translated into the corresponding amino-acid sequences. Next, these amino-acid sequences were evaluated using five HLA class I peptide-binding algorithms (NetMHC v3.2, NetMHCpan, PickPocket, SMM, SMMPMBEC) to identify neoantigens predicted to bind with high affinity to the patient's HLA alleles. As each algorithm prioritizes different binding characteristics, any mutation with an IC50 value of < 500 nm was considered a potential candidate neoantigen. We then set a criterion so that at least three of the five algorithms had to be concordant in predicting a binding score < 500 nM for a neoantigen to be considered for further in vitro analysis. Additionally, we evaluated the corresponding wild-type sequences to compare differences in predicted binding affinities.

### Peptide synthesis

**Murine peptides** Peptides were synthesized by Genscript (Piscataway, NJ, USA) with a purity of > 95%. Corresponding 25-mer peptides were synthesized based on the amino acid sequence predicted by algorithms. The 25-mer peptides were synthesized with mutations in a central position centered with approximately equal number of flanking amino acids before and after the mutation. Peptide structure and purity were verified by mass spectrometry and high-performance liquid chromatography (HPLC). Lyophilized peptides were stored at 4 °C with CaSO_4_ desiccant (Drierite) until needed for experiments, then dissolved in either DMSO or molecular grade water (as recommended by GenScript) at 20 mg/ml, aliquoted, and stored at –80 °C.

**Human peptides** Synthetic peptides were obtained from Peptide 2.0 (Chantilly, VA, USA) with a purity of > 95%. Peptide structure and purity were verified by mass spectrometry and high-performance liquid chromatography (HPLC). Peptides were dissolved in 4% DMSO and diluted with water to make 2 mM stock solutions.

### SLP vaccination

SLPs containing the identified neoantigens were custom-made by GenScript (Piscataway, NJ). Mice were vaccinated as previously detailed by our group [49].

### In vitro ELISPOT analysis

**Murine studies** A single cell suspension of splenocytes was created by passing the freshly harvested spleen through a 70-μm filter in CTL media (RPMI with 10% FBS, 0.5% l-glutamine, 1% penicillin/streptomycin, and 0.05 mM 2-mercaptoethanol). Erythrocytes were removed by RBC lysis (BioLegend) buffer, and the resulting splenocytes were washed and counted. Mouse IFN-y ELISpot^PLUS^ plates were conditioned with CTL media for 30 min at room temperature. The plates were then washed with PBS and plated with 200,000 splenocytes/200 µl/well. Splenocytes were pulsed with 4ug peptide in 100uL CTL media and were incubated for 24 h at 37 °C at 5% CO_2_. Cells were removed from the plate by washing 5 times with PBS. Wells were incubated for 2 h at room temperature with 1 μg/ml biotinylated anti-mouse IFN-γ mAb R4-6A2 (Mabtech) in 0.05% FBS diluted in PBS. Wells were washed as before, incubated with 1 μg/ml Streptavidin-ALP for 1 h at room temperature, and washed again. BCIP/NBT-plus substrate was added, and wells were developed for 10–15 min at room temperature. The reaction was stopped with tap water, and plates were allowed to dry for 24 h before they were counted using an automated image ELISPOT reader (ImmunoSpot).

**Human studies** Cryopreserved PBMCs were thawed and washed with serum-free media twice. PBMCs were plated at 2 × 10^5^ cells/well of a 96 well ELISpot plate pre-coated with human INF-γ (mAb 1-D1K, Mabtech, Cincinnati, OH, USA). Peptides were added to the wells at a final concentration of 25 µM. Peptide-pulsed PBMCs were incubated for 48 h at 37 °C at 5% CO_2_. INF-γ ELISpot plates were developed per the manufacturer's directions using biotinylated detection Ab (mAB 7-B6-1), Streptavidin-ALP, and BCIP/NBT-plus substrate. Spots were counted using an ELISpot reader (ImmunoSpot).

### Tumor protection studies

Mice (10/group) were vaccinated with SLP mixed with Poly ICLC or Poly ICLC alone on days 0 and 7.5 × 10^5^ tumor cells were inoculated subcutaneously in the flank of each mouse on Day 14. Tumor growths were measured every 3 days using electronic caliper.

### Statistical analysis

Data were plotted and analyzed using GraphPad Prism 8 (GraphPad, La Jolla, CA) and presented as mean ± SEM. Unpaired *T*-tests were used to compare between ICI treatment group with control. ANOVA tests, followed by Tuckey tests, were used to compare immune profiles between different clones. A *p*-value of less than 0.05 was considered statistically significant. To analyze tumor growth curves, the over-time change of tumor volumes were assessed by linear mixed model to account for the potential correlation among multiple measures taken from the same mouse, followed by post hoc multiple comparisons for between-group differences of interest. Logarithm transformation was performed to reduce data dispersion and to better satisfy normality assumption.

## Results

### Strategy to induce cancer neoantigens and clinically relevant models of PDAC

**Fig. 1:**
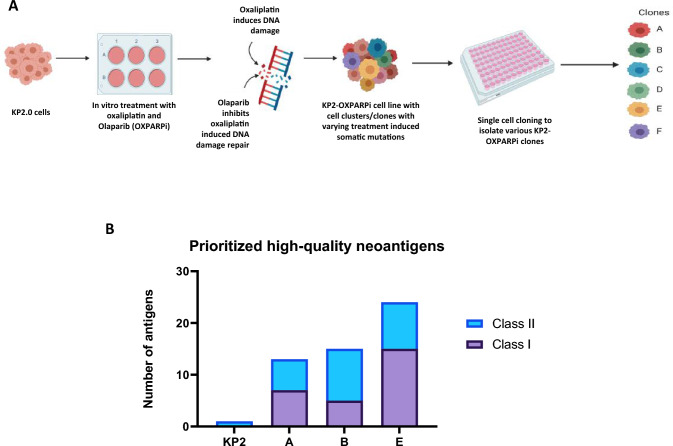
Treating KP2 cells with mutation inducing chemotherapies results in an increased expression of predicted neoantigens. **a** Schematic demonstrating the method of KP2-OXPARPi model development. KP2 cells were treated with oxaliplatin and Olaparib for multiple passages in a 6-well plate over the duration of four months. The cells count was performed for each well prior to cell passage. The resulting KP2-OXPARPi cell line was then used to perform single cell cloning. The cells were diluted in cell culture media so that 1 cell/100ul was added to each well in a 96well plate to isolate the clones from parental KP2-OXPARPi cell line. **b** In silico neoantigen prediction using pVAC-seq prioritized 1 class II neoantigen for parental KP2, compared to 7 and 6, 5 and 10, and 15 and 9 class I and II neoantigens for clones A, B, and E respectively with binding affinity < 1000 nM and RNA VAF > 0. This demonstrates OXPARPi treatment successfully increased tumor mutational burden and candidate neoantigen burden

The KP2 cell line was derived from the tumor of a KPC mouse [37, 38]. KP2 tumors have been previously shown to closely recapitulate the human PDAC immune microenvironment [10, 39, 50]. KP2 tumor cells were subjected to treatment with oxaliplatin and olaparib (OXPARPi) in vitro to induce somatic mutations (Fig. 1a). Oxaliplatin is platinum-based chemotherapy that exerts its cytotoxic effects by damaging genomic DNA by causing intra-strand and inter-strand crosslinks. It also has a mutagenic effect on DNA, inducing higher frequencies of small deletions/insertions and nucleotide transversions [51–53]. Olaparib is a poly (ADP-ribose) polymerase inhibitor (PARPi) that blocks the error-free process of homologous recombination repair, leading to genetic alterations and instability [54, 55]. Together, these two agents synergize to induce DNA damage. Upon completing treatment with oxaliplatin and olaparib, we utilized single-cell cloning by limiting dilution technique to isolate KP2-OXPARPi clones with unique mutational changes.

To evaluate the impact of OXPARPi on neoantigen burden, we performed exome sequencing and RNA-sequencing on the parental KP2 cell line, and clones A, B, and E. These clones were chosen to be a representation of ICI-sensitive and ICI-resistant clones (see next section). A high number of nonsynonymous missense and frameshift mutations were identified in the clones. In line with previously published data, the KP2 tumor line harbored a low mutational burden and only expressed a single class II neoantigen based on analyses using the pVAC-Seq suite of software tools (Fig. 1b). For expressed mutations, candidate 8–11 and 13–25 amino acid-long minimal peptides were analyzed using the pVAC-tools suite of epitope prediction algorithms [56]. 13, 15, and 24 neoantigens were predicted for KP2-OXPARPi clone A, B and E respectively (Fig. 1b, Supplementary Table 1). In contrast to only 1 neoantigen was predicted for the parental KP2 cell line, our results confirm that OXPARPi treatment followed by cloning can generate cell lines with novel neoantigen profiles.

### Combination immune checkpoint inhibition significantly slows the tumor growth of select KP2-OXPARPi tumors

**Fig. 2:**
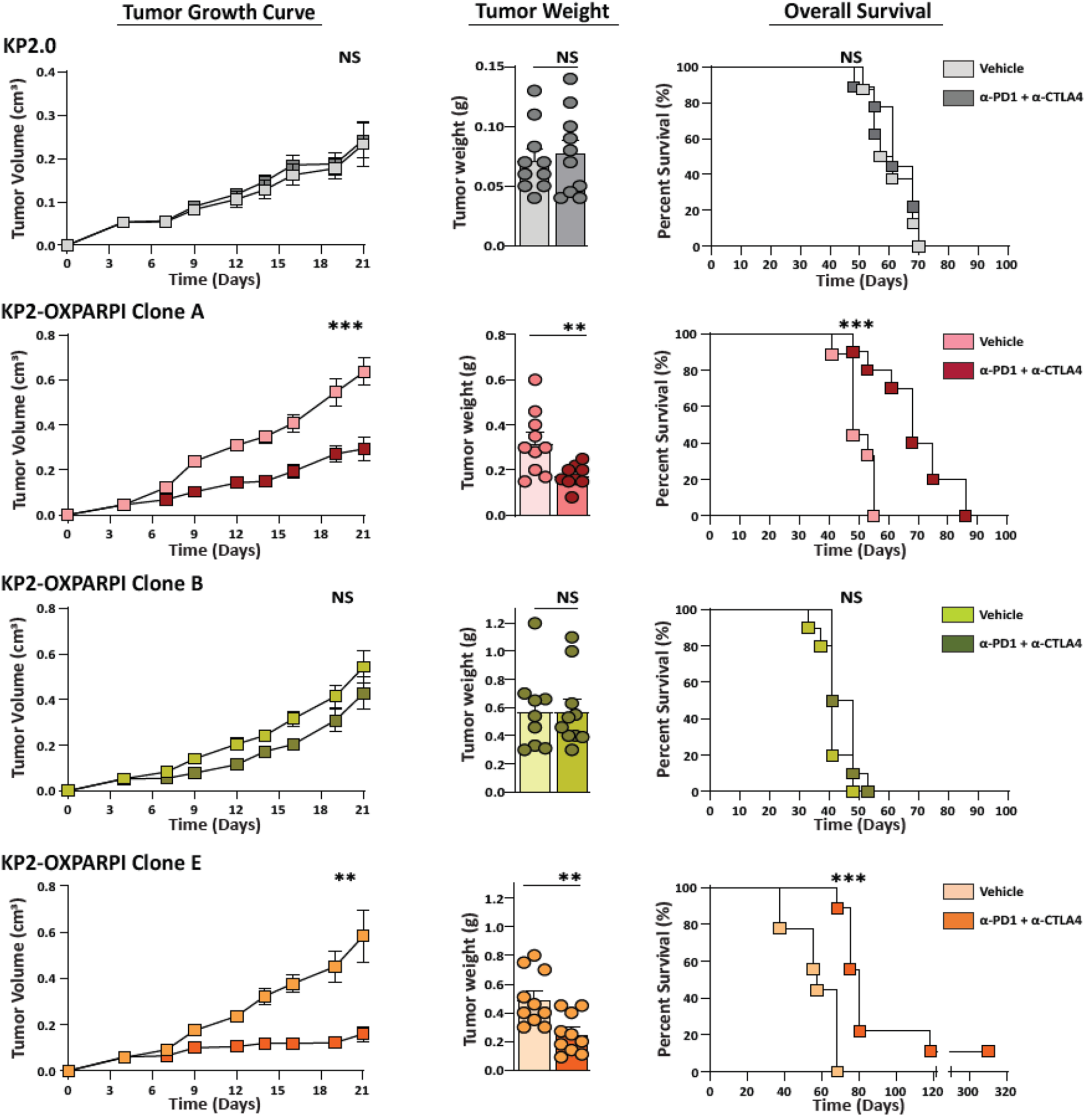
Tumors derived from KP2-OXPARPi clone A and E are responsive to ICI therapy with anti-PD1 and anti-CTLA4, while Clone B is resistant. Tumor growth in subcutaneous models of KP2-OXPARPi clones A, B, and E shown by 21-day tumor volume curves, Day 21 tumor weight, and survival curves (*N* = 9–10/group). Mice were implanted with 5 × 10^5^ cells subcutaneously. On Day 5, tumor volume was measured, and mice were randomized into vehicle and treatment groups. Treatment group mice were administered 200ug/dose of anti-PD1 every 3-days, and 250 µg/dose of anti-CTLA4 every 4 days. *p < 0.05, **p < 0.01, ***p < 001. NS = Not Significant

To determine ICI sensitivity of the OXPARPi clones, we treated the parental KP2 cell line and KP2-OXPARPi clones with immune checkpoint inhibitors in vivo. KPC mouse-derived PDAC models are not typically susceptible to immune checkpoint blockade therapies, unlike carcinogen-driven sarcoma models [7, 27, 39]. However, we observed a significantly reduced progression in tumor growth in KP2-OXAPRPi clones A (*p* = 0.0005), D (*p* = 0.03), and E (*p* = 0.0003) tumors over the 16 days of treatment (Fig. 2, and Supplemental Fig. 2). This short-term tumor control was sustained even after the cessation of treatment and resulted in increased survival (clone A—*p* = 0.0007, clone D—*p* = 0.0034, and clone E—*p* = 0.0001) in mice treated with ICI. Two mice in clone E group became tumor free between days 40 and 45 and survived past 120 days. In contrast, combination ICI treatment did not significantly affect tumor growth of clones B, C, and F, and our experiments confirmed that parental KP2 tumors are ICI-resistant as well.

### ICI-sensitive KP2-OXPARPi clones A and E exhibit different immune infiltrate and gene expression compared to ICI-resistant clone B and parental KP2

**Fig. 3:**
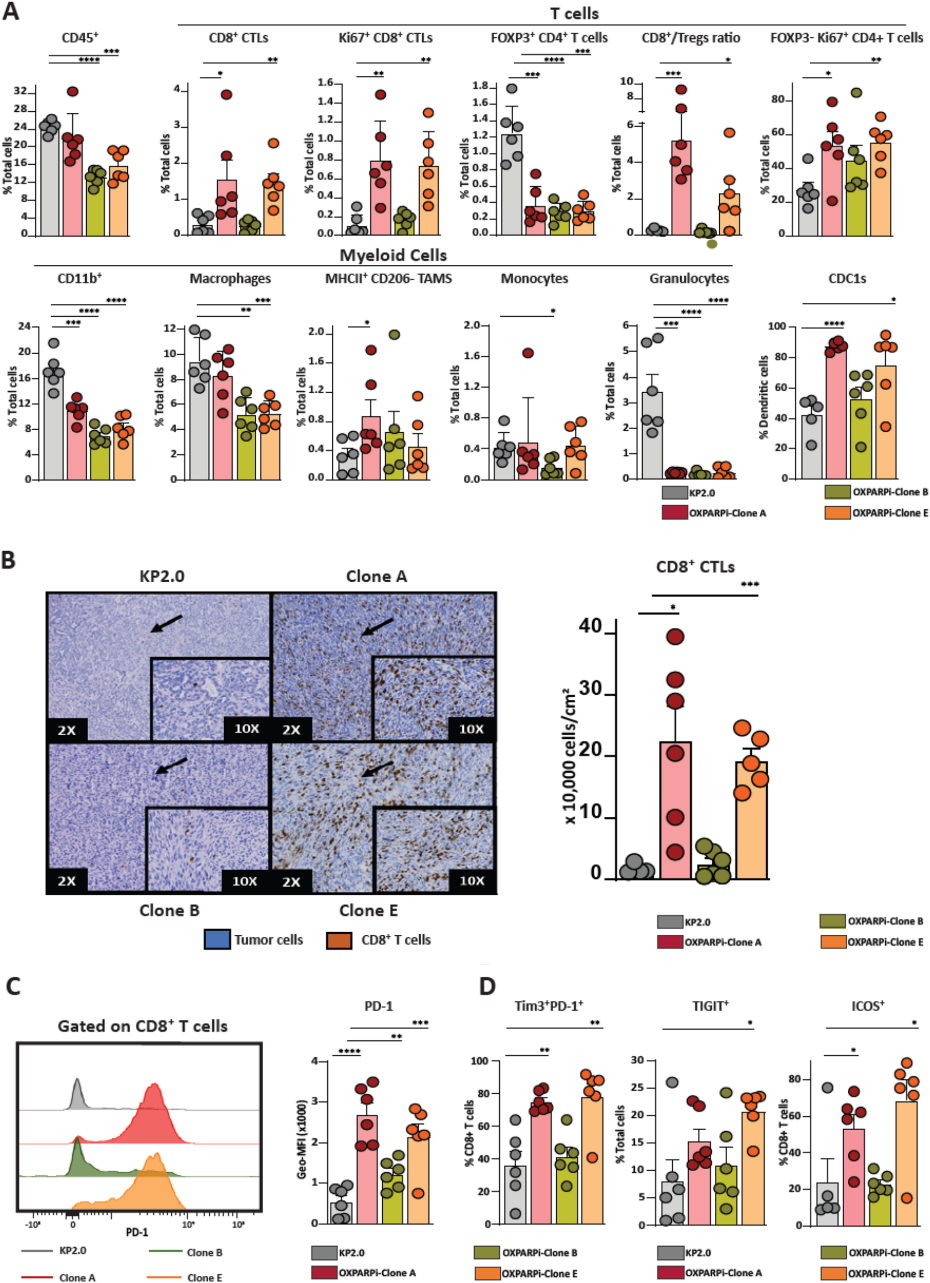
Characterization of tumor immune infiltrate of KP2-OXPARPi tumors. **a** Quantification of CD45^+^ immune cells into CD11b^+^, macrophages (TAMs), monocytes, granulocytes, CD8^+^ T cells, Ki67^+^ CD8^+^ T cells, FOXP3^+^ CD4^+^ T cells, CD8^+^ Tcells/Tregs ratio, FOXP3^−^ Ki67^+^ CD4^+^ T cells and conventional dendritic cells type 1 (cDC1s) (*N* = 5–6/group). B) Representative IHC images (2X and 10X) showing the quantification of CD8 + T cell population in KP2 and KP2-OXPARPi tumors (*n* = 4–6/group). Graphs showing the number of CD8^+^T cells per cm^2^ of tumor. Frequencies and phenotypes of tumor-infiltrating CD8^+^ T cells in KP2-OXPARPi tumors (*n* = 5–6/group). **c** Baseline PD-1 expression on KP2-OXPARPi tumor lines is shown as histograms of geometric mean fluorescent intensity (Geo-MFI) on CD8 + T cells (*N* = 6/group). *p < 0.05, **p < 0.01, ***p < 001, ****p < 0.0001

To understand the immunobiology of OXPAPRi clones, we analyzed the immune infiltrates of clones A, B, E, and the parental KP2 line as representative ICI-sensitive and ICI-resistant tumors. Clones A and E demonstrated a significantly higher number of total CD8 T cells than parental KP2 tumors (KP2 vs. Clone A, *p* = 0.04; KP2 vs. Clone E, *p* = 0.0019) (Fig. 3a). In addition to an increased T cell frequency, clones A and E also showed an increased proportion of actively dividing CD8 and CD4 effector T cells (Ki67^+^/CD8^+^ T cells, Ki67^+^/FOXP3^−^/CD4^+^ T cells). We also noted significantly less FOXP3^+^ regulatory CD4^+^ T cells and an increase in the ratio of CD8 T cells to regulatory T cells**.** IHC confirmed a similar increase in total CD8 T cell numbers (Fig. 3b). Furthermore, we observed an increase in PD-1^+^, PD-1^Hi^/Tim-3^Hi^, and TIGIT^+^ CD8^+^ T cells, suggesting that the infiltrating effector T cells had a more exhausted phenotype (Fig. 3c, d). We also noted an increase in expression of co-stimulatory molecule ICOS on CD8 T cells. Interestingly, clone B tumors did not follow a similar trend and demonstrated minimal T cell infiltration, proliferation, and activation, like the parental KP2 tumors.

When comparing the myeloid cell infiltrate of KP2-OXPARPi clonal tumors to KP2 tumors, we observed a significant decrease in the total CD11b^+^ population in KP2-OXPARPi tumors (Fig. 3a). We also noted a significant decrease in macrophage population in clones B and E tumors. While the total macrophage population in clone A was similar to KP2 tumors, there is a significant increase in the antigen presenting MHC-II^high^ macrophage subset. We also found a significant decrease in granulocytes infiltration that was consistent across all clones. Monocyte infiltration was significantly decreased in clone B tumors. The tumor-infiltrating DCs in clones A and E tumors were predominantly cDC1s which play an important role in CD8^+^ T cell-mediated anti-tumor immunity [57, 58].

RNA-sequencing demonstrated significant gene expression differences between clones A and E, and clone B and parental KP2 line (Supplementary Fig. 1A). For example, expression of genes involved in antigen processing and presentation was markedly higher in clones A and E compared to clone B and parental KP2. Furthermore, numerous pathways involved in T-cell development, checkpoint inhibition, chemokine signaling, and inflammation were enriched in clones A and E compared to clone B (Supplementary Fig. 1B). Additionally, expression of chemokines involved in T cell recruitment (e.g. CCL5), and PD-L1 (CD274) was much higher in the clones A and E compared to clone B and parental KP2. Taken together, these studies suggest KP2-OXPARPi clones A and E, and clone B and parental KP2 represent two distinct immunologic phenotypes that correlate with their ICI-sensitivity.

### T cells contribute to ICI-mediated restraint of tumor growth

**Fig. 4:**
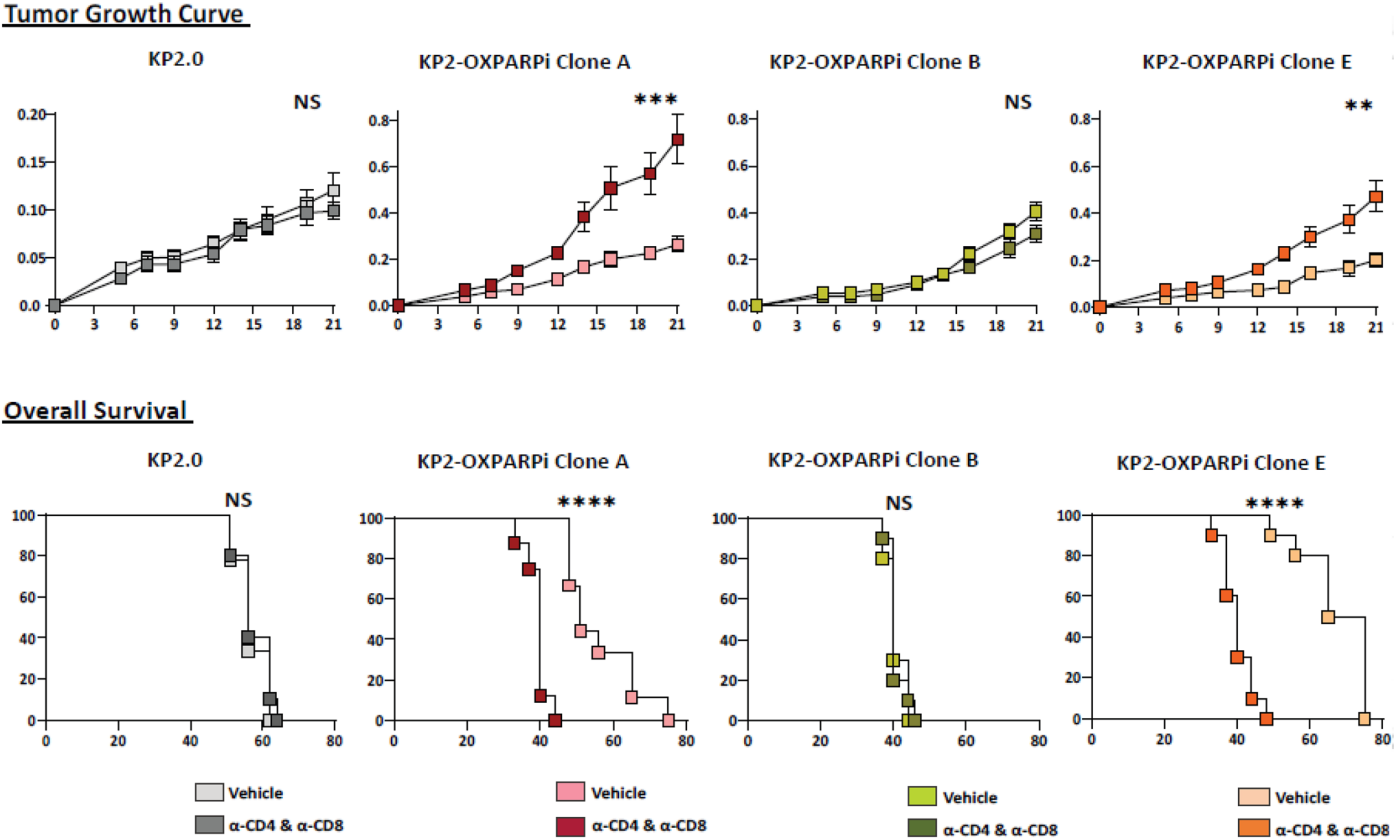
KP2-OXPARPi A and E tumors grow in a T-cell dependent manner A, while B does not. Tumor growth in subcutaneous models of KP2-OXPARPi clones A, B, and E shown by 21-day tumor volume curves, Day 21 tumor weight, and survival curves (*N* = 9–10/group). Mice were implanted with 2.5 × 105 cells subcutaneously. On Day 5, tumor volume was measured, and mice were randomized into vehicle and treatment groups. Treatment group mice were administered 250 µg/dose of anti-CD4 and anti-CD8 depletion IgGs every 4-days. *p < 0.05, **p < 0.01, ***p < 001. NS-Not Significant

To determine whether the presence of relatively high numbers of proliferating and activating CD8 and CD4 T cells has an effect on the progression of KP2-OXPARPi tumors, we depleted CD4 and CD8 T cells using CD4 and CD8 depleting antibodies to KP2-OXPARPi tumor-bearing mice. The efficacy of antibody-mediated T cell depletion was confirmed by FACS analysis (Supplementary Fig. 2C). In immunologically active clones A and E, CD4^+^ and CD8^+^ T cell depletion resulted in faster growth of tumors and worse survival compared to controls (Fig. 4). This contrasts with clone B and parental KP2 tumors for which T cell depletion did not alter tumor growth kinetics and overall survival.

### Targeting prioritized neoantigens in KP2-OXPARPi clone E restrains tumor growth

**Fig. 5:**
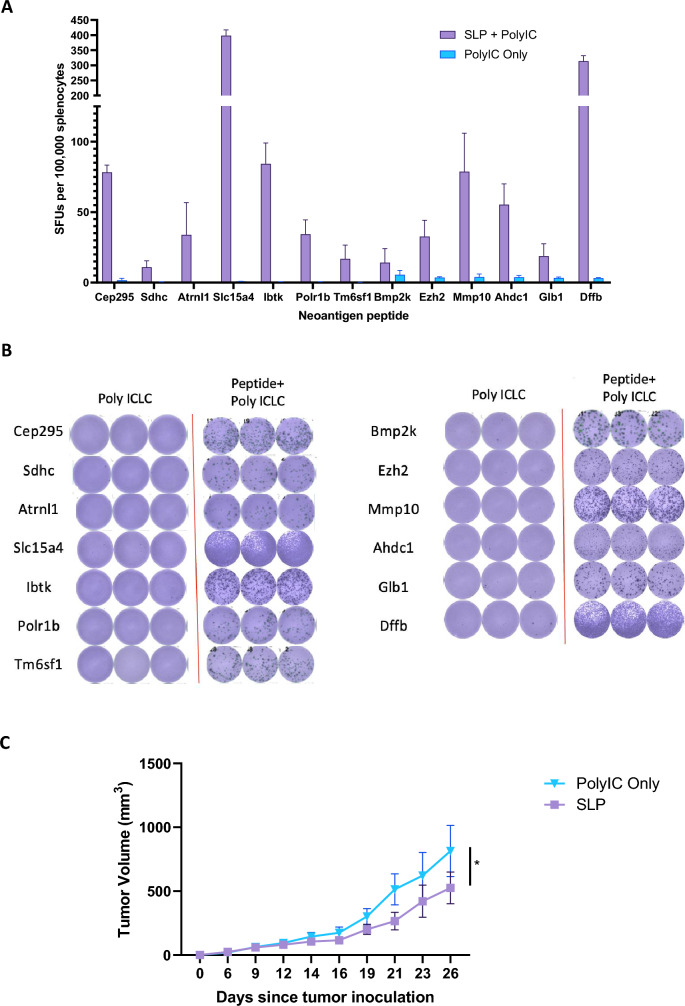
Neoantigen reprioritization based on RNA-seq and vaccination screening studies. **a** Out of 24 predicted neoantigens for clone E, 13 were validated to be immunogenic through vaccination studies which generated a T cell response measured by IFN-ϒ ELISpot assay. Mice (*n* = 5/group) were vaccinated with Poly ICLC alone or Poly ICLC + neoantigen peptides on days 0, and 7. The spleens were harvested on Day 13 and ELISpot assay was performed. **b** Sample ELISpot wells from vaccination experiments. Data are combined from multiple separate experiments. Wells shown are representative triplicates or duplicates of a single mouse. **c** SLP vaccination with 13 immunogenic neoantigens for clone E in the prophylactic setting restrains tumor growth after subcutaneous inoculation

To assess immunogenicity and induction of antitumor immunity of predicted neoantigens, we selected predicted neoantigen epitopes for clone E with IC50 < 1000 nm and RNA VAF > 0 for validation through immunization studies. Mice vaccinated with synthetic long peptide (SLP) vaccines demonstrated a T cell response measured by IFN-ϒ ELISPOT to 13 out of 24 neoantigens (Fig. 5a and b). A 2-dose SLP vaccine containing the 13 immunogenic neoantigens in the prophylactic setting significantly inhibited growth of KP2-OXPARPi clone E in vivo compared to control (*p* = 0.0157) (Fig. 5c).

### Number and quality of KP2-OXPARPi neoantigens mirror human PDAC neoantigens

There are currently many different strategies to identify cancer neoantigens with highly variable results (for example, please see the detailed comparisons of different neoantigen vaccine strategies in [59]). Given the highly variable results of these different strategies, we thought it was important to compare the number and quality of cancer neoantigens of our mouse model to the number and quality of cancer neoantigens in human pancreatic cancer patients using the same neoantigen prediction strategy.

We collected tumor samples from three consecutive patients undergoing surgical resection for pancreatic cancer at our center. Specimen acquisition, quality control, and nucleic acid isolation protocols are described in the Methods section (Supplementary Fig. 4A). Despite the concerns of low cellularity, dense desmoplastic stroma, and overexpression of RNAse in PDAC, we were able to obtain sufficiently high-quality samples for exome sequencing [60–62]. Nucleic acid integrity was determined using RNA integrity numbers (RIN) and fragment distribution values (DV200). RIN scores were relatively low, with a mean score of 2.35 (range: 2.3–2.4), reflecting that RNA was isolated from FFPE tissues. The mean DV200 was 59% (range 32–72%), with one sample falling below 40%. RNA libraries were successfully constructed and sequenced from all samples.

Following the confirmation of nucleic acid integrity, we proceeded to tumor exome and cDNA-capture sequencing to identify somatic mutations (Supplementary Fig. 5A). A KRAS^G12V^ driver mutation was identified in all three specimens. The median KRAS DNA variant allele frequency (VAF), which was used as a surrogate for tumor purity, was 32% (range 20–64%) (Supplementary Fig. 5B). One sample had a loss of heterozygosity at the mutated KRAS locus. The median number of nonsynonymous mutations identified was 31 (range: 18–34; DNA VAF cutoff 10%). Of these, a median of 9 mutations (range: 7–10) were expressed at the mRNA level using an RNA VAF cutoff > 10% and Fragments Per Kilobase of transcript per Million mapped reads (FPKM) > 1**.**

The pVAC-Seq suite of computational tools was used to predict if the nonsynonymous mutations expressed in tumors were likely to bind the patient’s MHC-I alleles with high affinity. In our analysis, a median of 5 candidate neoantigens (range: 4–6) were predicted to bind to the patient's MHC-I alleles with a binding affinity < 500 nM. As others have shown that the difference in binding affinity between the mutant and wild-type peptide may be important for recognizing neoantigens, we also assessed the fold change in binding affinity [63]. Of the 16 neoantigens identified across three patients, 7 neoantigens had a fold change of > 10 by at least one binding algorithm. However, there was less concordance in the fold change score between the algorithms (Supplementary Fig. 4B). An analysis of the mutant amino acid position showed that across the three patients, 14 of the 16 mutations that created neoantigens were due to mutations in predicted MHC-I anchor residues.

The prevalence of pre-existing immune responses to candidate neoantigens was determined by performing in vitro assays. The patient's PBMCs were cultured for 48 h with synthetic peptides corresponding to the candidate neoantigens, followed by an IFN-γ ELISpot assay. Interestingly, we did not see a correlation between the predicted neoantigen binding score, and the pre-existing immune response detected by ELISPOT. As shown in Supplementary Fig. 4C, a pre-existing response was identified to at least one candidate neoantigen in each patient (Subject A—SDHA, 8117—Subject B—ZRANB1, Subject C—ATAD3C). These findings suggest that when the same neoantigen identification strategy is applied, the total number of candidate neoantigens, and quality of the neoantigens in the KP2-OXPARPi clones mirror what is observed in human PDAC patients.

## Discussion

In order to develop a preclinical mouse model of PDAC that expresses neoantigens at a similar frequency as human PDAC, we utilized a novel strategy of treating the KPC-derived cell line KP2 with oxaliplatin and olaparib in vitro to induce somatic mutations. The reason for selecting oxaliplatin and olaparib to develop our preclinical model was based on their clinical relevance in PDAC management. Oxaliplatin is a platinum-based chemotherapy that is a component of FOLFIRINOX, a first-line combination therapy for locally advanced and metastatic PDAC [64]. Like other platinum-based drugs, oxaliplatin can cause dose-dependent somatic mutations [51, 52]. However, unlike cisplatin and carboplatin, oxaliplatin retains its mutagenic properties even at low drug concentrations [53]. Rumble et al. recently showed that treating a colorectal cancer line, HCT116, with low-dose oxaliplatin caused somatic mutations and led to the expression of neoantigens [65]. However, most cancer cells eventually develop resistance against oxaliplatin, mainly by overexpressing enzymes that are involved in mismatch repair (MMR) and nucleotide excision repair (NER) [66]. Therefore, we decided to combine oxaliplatin with a small molecule inhibitor that can counteract this mechanism of resistance. Olaparib is a PARP inhibitor that inhibits the repair of single-strand breaks (SSBs) in cancer cells, which subsequently results in the accumulation of double-strand breaks (DSBs) [55]. It has been effective in tumors, including pancreatic cancer, with deficient homologous repair due to BRCA1/2 mutations, where DNA breaks are repaired by more error-prone pathways such as non-homologous end joining, resulting in cell cycle arrest, genetic instability, and cell death [67–69].

By treating KP2 cells with a low concentration of oxaliplatin and olaparib, we aimed to induce somatic mutations and inhibit their subsequent repair, ensuring that the treated tumor line will express these mutations with the goal of generating genetic diversity. Using two synergistic agents allowed us to use lower doses and avoid drug toxicity or resistance. Since the induced mutations were expected to occur as random genetic events at different time points during the treatment, we anticipated that the treated tumor line would consist of multiple subpopulations/clones with distinct mutational changes. Therefore, we performed single-cell cloning to isolate six clones from the treated KP2-OXPARPi cell line.

Like human PDAC, KPC tumor immune infiltrate is characterized by the abundance of immunosuppressive myeloid cells and minimal tumor-infiltrating T cells, which seldom interact with malignant cells and play a negligible role in tumor development [38, 70]. In our tumor model KP2-OXPARPi clones A and E demonstrated increased T cell infiltration compared to clone B and the parental KP2 line. The degree of T cell infiltration is associated with immune responsiveness in vivo.

Identification of candidate neoantigens is a complex bioinformatics task. Here, we utilized pVAC-tools, an open-source suite of neoantigen prediction tools, to identify and prioritize neoantigens in KP2-OXPARPi clones [43, 56]. pVAC-tools uses a set of seven MHC-I and three MHC-II prediction algorithms, as described in the Methods section. While the MHC class I neoantigen prediction algorithms are stringent/reliable due to the small variation in epitope length (8–11 amino acids in length), the process of the MHC-II epitope prediction is complicated by the variations in peptide length (13–25 amino acid in length) due to the open structure of MHC-II molecule binding groove, identification of the correct binding core, and the effect of peptide flanking regions on peptide immunogenicity [71–76]. Despite these factors, studies have shown that vaccinating against MHC-II neoantigens that were identified using current prediction algorithms is therapeutically effective in both preclinical and clinical settings [8, 13, 14]. Similarly, Schreiber et al. recently demonstrated the functional role of MHC-II tumor neoantigens in mediating checkpoint immunotherapy dependent anti-tumor immune response in their murine sarcoma model [10]. In our model, we have successfully identified both MHC-I and MHC-II neoantigens in KP2-OXPARPi clones A, B, and E which are absent in the parental KP2 cell line. Through vaccination studies, we demonstrated that a subset of MHC class I and II neoantigens was able to generate T cell responses as measured by ELISPOT analysis. Vaccination with these immunogenic neoantigens can restrain tumor growth.

In our patient cohort, we applied the same neoantigen prediction strategy used for the KP2-OXPARPi clones and identified cancer neoantigens in all three patients. We felt using the same prediction model for both the murine and human samples would be an important validation given the highly variable results from different prediction strategies [59]. A strong pre-existing immune response to at least one neoantigen was documented in each patient. Demonstration of pre-existing immune responses supports the hypothesis that neoantigens can generate anti-tumor immune responses in humans, even in intermediate mutation burden tumors like PDAC. This intermediate mutation burden and resulting low number of neoantigens is recapitulated in the KP2-OXPARPi model we have generated.

Based on these results, we have initiated phase I clinical trials to assess the safety and immunogenicity of a neoantigen DNA and peptide vaccination strategy in PDAC patients (NCT03122106 and NCT03956056, respectively). Patients are treated with vaccination in the adjuvant setting following surgery and adjuvant chemotherapy [77]. Although these patients are at high risk for disease recurrence, treatment in the adjuvant setting provides a window for sequencing and vaccine manufacture. To move this strategy into patients with existing disease will likely require combining neoantigen vaccination with immune-modulatory therapies focused on overcoming the immunosuppressive PDAC infiltrate [39–41, 78].

Limitations of our model highlight our incomplete understanding of the immunobiology of pancreatic cancer, and specifically the role of cancer neoantigens in antitumor immune responses. While we have confirmed a paucity of neoantigens in the parental KP2 line and presence of a relevant number of cancer neoantigens in the KP2-OXPARPi clones, the exact role of these neoantigens in anti-tumor immunity is unclear. The mechanism(s) of antitumor immune responses in the KP2-OXPARPi clones is likely multifactorial and currently, the role of neoantigens is supported only by indirect evidence. While it is uncertain to what degree the KP2-OXPARPi neoantigens recapitulate neoantigens in human PDAC, we have introduced neoantigens to a highly relevant genetic model. We hope future studies exploring the roles of neoantigens, T-cell infiltration, and antigen processing and presentation in our model will provide important insights into human pancreatic cancer.

The KP2-OXPARPi model described here, provide a number of clones with varying tumor microenvironments, degree of T cell infiltration, and ICI-sensitivity. Furthermore, we have successfully generated cancer neoantigens which are capable of restraining tumor growth. The KP2-OXPARPi clones could thus serve as a model for future investigations in the immunobiology and role of cancer neoantigens in pancreatic cancer.

### Supplementary Information

Below is the link to the electronic supplementary material.

Supplementary file1 (PPTX 1240 KB)

Supplementary file2 (DOCX 22KB)

## Data Availability

The raw genetic sequencing data files analyzed during the current study are available from the corresponding author on reasonable request. The remaining data generated and analyzed during this study are included in the article and supplementary files. All software packages used are publicly available through commercial vendors.

## Declarations

### Human or animal participants

Animal protocols were approved by the Animal Studies Committee of Washington University School of Medicine and were in accord with IACUC guidelines and procedures. This study was performed in accordance with the Declaration of Helsinki.
