# Supplementary material for: Induction of cancer neoantigens facilitates development of clinically relevant models for the study of pancreatic cancer immunobiology
Source: Cancer Immunol Immunother. 2023 May 13;72(8):2813–27. doi: 10.1007/s00262-023-03463-x (PMC10361914; doi:10.1007/s00262-023-03463-x)
Supplement: Supplementary file 1 — Supplementary file1 (PPTX 1240 KB) [file 262_2023_3463_MOESM1_ESM.pptx]

## Slide 1
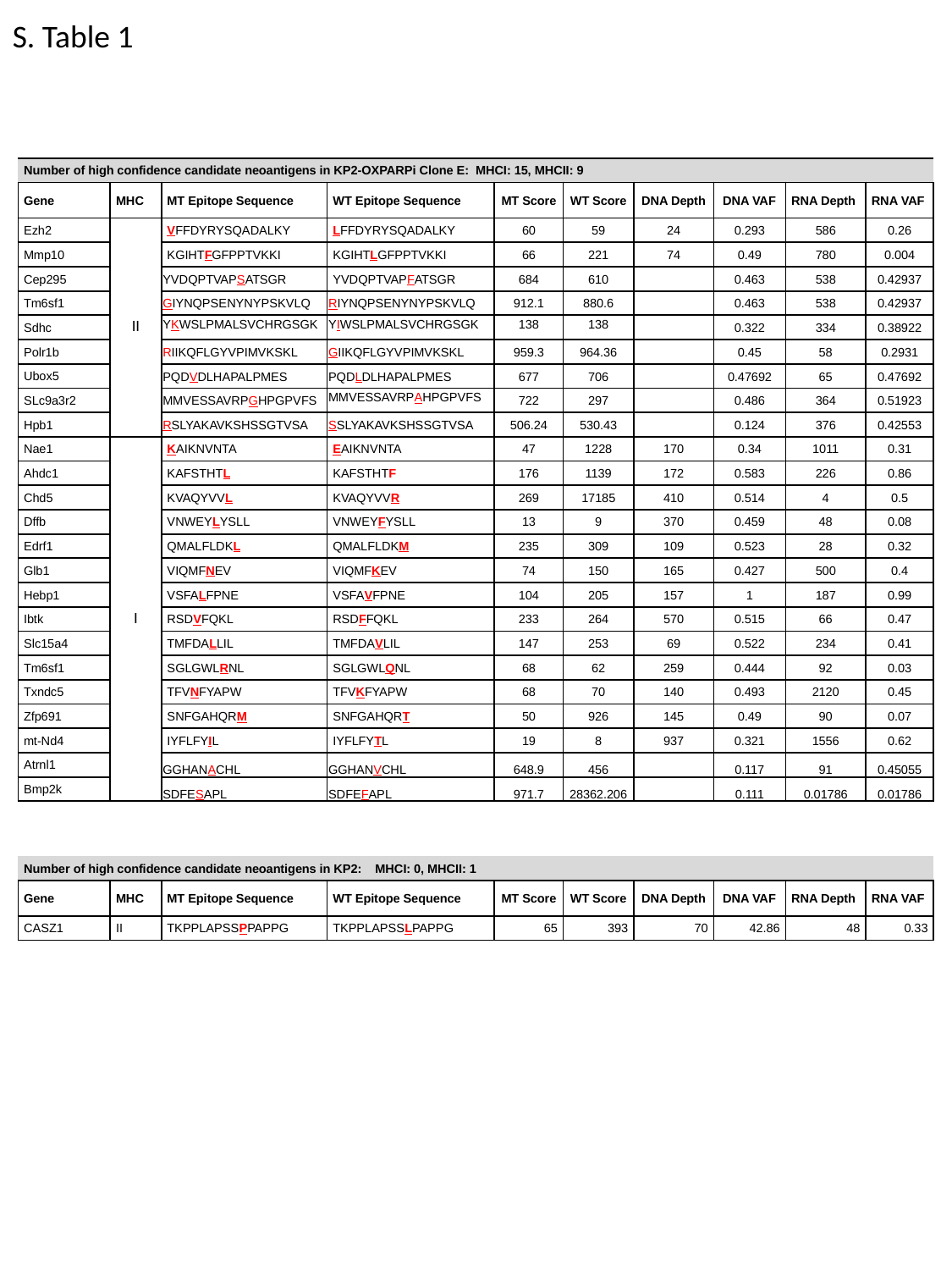

S. Table 1
| Number of high confidence candidate neoantigens in KP2-OXPARPi Clone E: MHCI: 15, MHCII: 9 | | | | | | | | | |
| --- | --- | --- | --- | --- | --- | --- | --- | --- | --- |
| Gene | MHC | MT Epitope Sequence | WT Epitope Sequence | MT Score | WT Score | DNA Depth | DNA VAF | RNA Depth | RNA VAF |
| Ezh2 | II | VFFDYRYSQADALKY | LFFDYRYSQADALKY | 60 | 59 | 24 | 0.293 | 586 | 0.26 |
| Mmp10 | | KGIHTFGFPPTVKKI | KGIHTLGFPPTVKKI | 66 | 221 | 74 | 0.49 | 780 | 0.004 |
| Cep295 | II | YVDQPTVAPSATSGR | YVDQPTVAPFATSGR | 684 | 610 | | 0.463 | 538 | 0.42937 |
| Tm6sf1 | II | GIYNQPSENYNYPSKVLQ | RIYNQPSENYNYPSKVLQ | 912.1 | 880.6 | | 0.463 | 538 | 0.42937 |
| Sdhc | II | YKWSLPMALSVCHRGSGK | YIWSLPMALSVCHRGSGK | 138 | 138 | | 0.322 | 334 | 0.38922 |
| Polr1b | II | RIIKQFLGYVPIMVKSKL | GIIKQFLGYVPIMVKSKL | 959.3 | 964.36 | | 0.45 | 58 | 0.2931 |
| Ubox5 | | PQDVDLHAPALPMES | PQDLDLHAPALPMES | 677 | 706 | | 0.47692 | 65 | 0.47692 |
| SLc9a3r2 | | MMVESSAVRPGHPGPVFS | MMVESSAVRPAHPGPVFS | 722 | 297 | | 0.486 | 364 | 0.51923 |
| Hpb1 | | RSLYAKAVKSHSSGTVSA | SSLYAKAVKSHSSGTVSA | 506.24 | 530.43 | | 0.124 | 376 | 0.42553 |
| Nae1 | I | KAIKNVNTA | EAIKNVNTA | 47 | 1228 | 170 | 0.34 | 1011 | 0.31 |
| Ahdc1 | | KAFSTHTL | KAFSTHTF | 176 | 1139 | 172 | 0.583 | 226 | 0.86 |
| Chd5 | | KVAQYVVL | KVAQYVVR | 269 | 17185 | 410 | 0.514 | 4 | 0.5 |
| Dffb | | VNWEYLYSLL | VNWEYFYSLL | 13 | 9 | 370 | 0.459 | 48 | 0.08 |
| Edrf1 | | QMALFLDKL | QMALFLDKM | 235 | 309 | 109 | 0.523 | 28 | 0.32 |
| Glb1 | | VIQMFNEV | VIQMFKEV | 74 | 150 | 165 | 0.427 | 500 | 0.4 |
| Hebp1 | | VSFALFPNE | VSFAVFPNE | 104 | 205 | 157 | 1 | 187 | 0.99 |
| Ibtk | | RSDVFQKL | RSDFFQKL | 233 | 264 | 570 | 0.515 | 66 | 0.47 |
| Slc15a4 | | TMFDALLIL | TMFDAVLIL | 147 | 253 | 69 | 0.522 | 234 | 0.41 |
| Tm6sf1 | | SGLGWLRNL | SGLGWLQNL | 68 | 62 | 259 | 0.444 | 92 | 0.03 |
| Txndc5 | | TFVNFYAPW | TFVKFYAPW | 68 | 70 | 140 | 0.493 | 2120 | 0.45 |
| Zfp691 | | SNFGAHQRM | SNFGAHQRT | 50 | 926 | 145 | 0.49 | 90 | 0.07 |
| mt-Nd4 | | IYFLFYIL | IYFLFYTL | 19 | 8 | 937 | 0.321 | 1556 | 0.62 |
| Atrnl1 | | GGHANACHL | GGHANVCHL | 648.9 | 456 | | 0.117 | 91 | 0.45055 |
| Bmp2k | | SDFESAPL | SDFEFAPL | 971.7 | 28362.206 | | 0.111 | 0.01786 | 0.01786 |
| Number of high confidence candidate neoantigens in KP2: MHCI: 0, MHCII: 1 | | | | | | | | | |
| --- | --- | --- | --- | --- | --- | --- | --- | --- | --- |
| Gene | MHC | MT Epitope Sequence | WT Epitope Sequence | MT Score | WT Score | DNA Depth | DNA VAF | RNA Depth | RNA VAF |
| CASZ1 | II | TKPPLAPSSPPAPPG | TKPPLAPSSLPAPPG | 65 | 393 | 70 | 42.86 | 48 | 0.33 |

## Slide 2
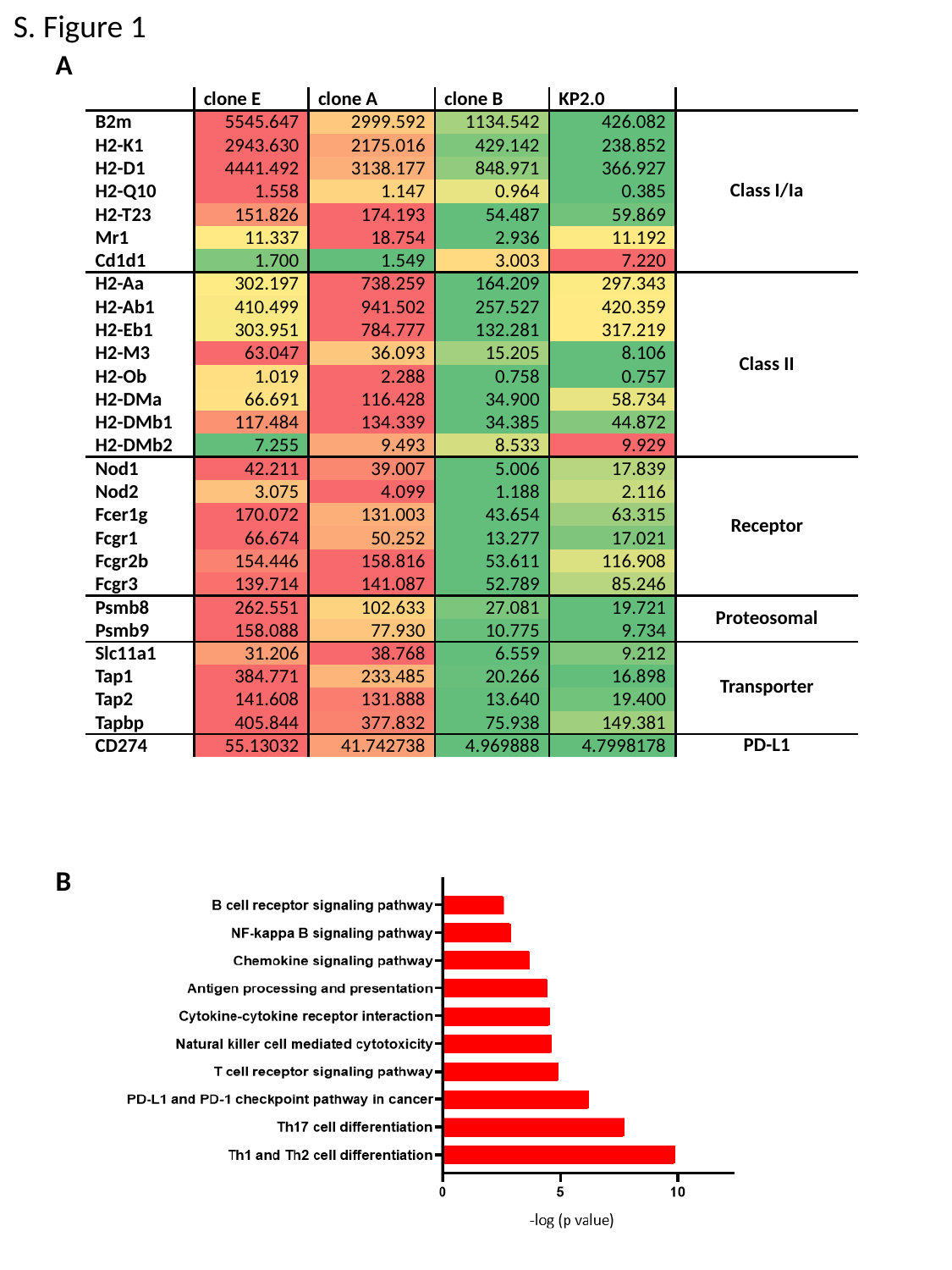

S. Figure 1
A
| | clone E | clone A | clone B | KP2.0 | |
| --- | --- | --- | --- | --- | --- |
| B2m | 5545.647 | 2999.592 | 1134.542 | 426.082 | Class I/Ia |
| H2-K1 | 2943.630 | 2175.016 | 429.142 | 238.852 | |
| H2-D1 | 4441.492 | 3138.177 | 848.971 | 366.927 | |
| H2-Q10 | 1.558 | 1.147 | 0.964 | 0.385 | |
| H2-T23 | 151.826 | 174.193 | 54.487 | 59.869 | |
| Mr1 | 11.337 | 18.754 | 2.936 | 11.192 | |
| Cd1d1 | 1.700 | 1.549 | 3.003 | 7.220 | |
| H2-Aa | 302.197 | 738.259 | 164.209 | 297.343 | Class II |
| H2-Ab1 | 410.499 | 941.502 | 257.527 | 420.359 | |
| H2-Eb1 | 303.951 | 784.777 | 132.281 | 317.219 | |
| H2-M3 | 63.047 | 36.093 | 15.205 | 8.106 | |
| H2-Ob | 1.019 | 2.288 | 0.758 | 0.757 | |
| H2-DMa | 66.691 | 116.428 | 34.900 | 58.734 | |
| H2-DMb1 | 117.484 | 134.339 | 34.385 | 44.872 | |
| H2-DMb2 | 7.255 | 9.493 | 8.533 | 9.929 | |
| Nod1 | 42.211 | 39.007 | 5.006 | 17.839 | Receptor |
| Nod2 | 3.075 | 4.099 | 1.188 | 2.116 | |
| Fcer1g | 170.072 | 131.003 | 43.654 | 63.315 | |
| Fcgr1 | 66.674 | 50.252 | 13.277 | 17.021 | |
| Fcgr2b | 154.446 | 158.816 | 53.611 | 116.908 | |
| Fcgr3 | 139.714 | 141.087 | 52.789 | 85.246 | |
| Psmb8 | 262.551 | 102.633 | 27.081 | 19.721 | Proteosomal |
| Psmb9 | 158.088 | 77.930 | 10.775 | 9.734 | |
| Slc11a1 | 31.206 | 38.768 | 6.559 | 9.212 | Transporter |
| Tap1 | 384.771 | 233.485 | 20.266 | 16.898 | |
| Tap2 | 141.608 | 131.888 | 13.640 | 19.400 | |
| Tapbp | 405.844 | 377.832 | 75.938 | 149.381 | |
| CD274 | 55.13032 | 41.742738 | 4.969888 | 4.7998178 | PD-L1 |
B

## Slide 3
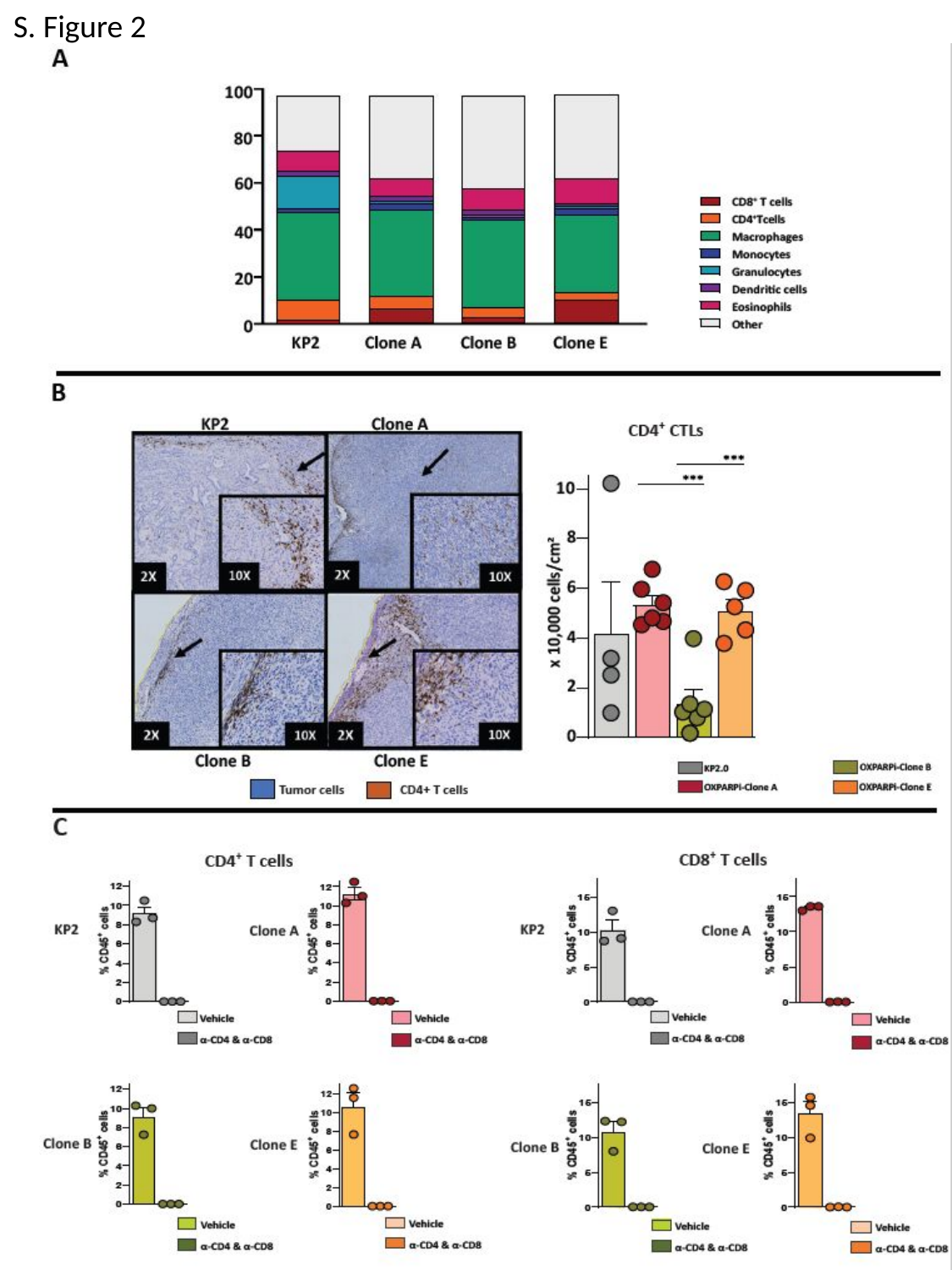

S. Figure 2

## Slide 4
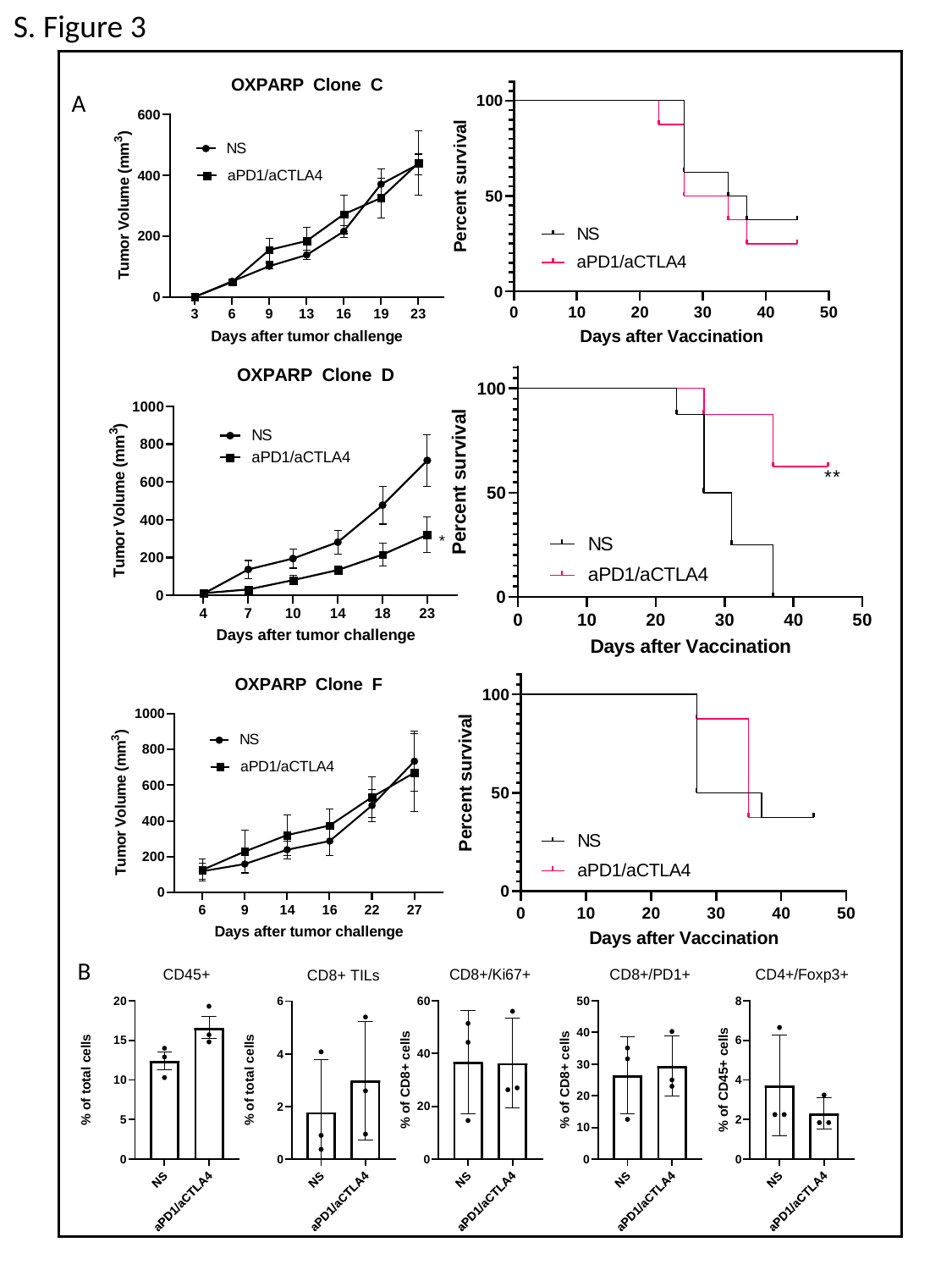

S. Figure 3
A
B

## Slide 5
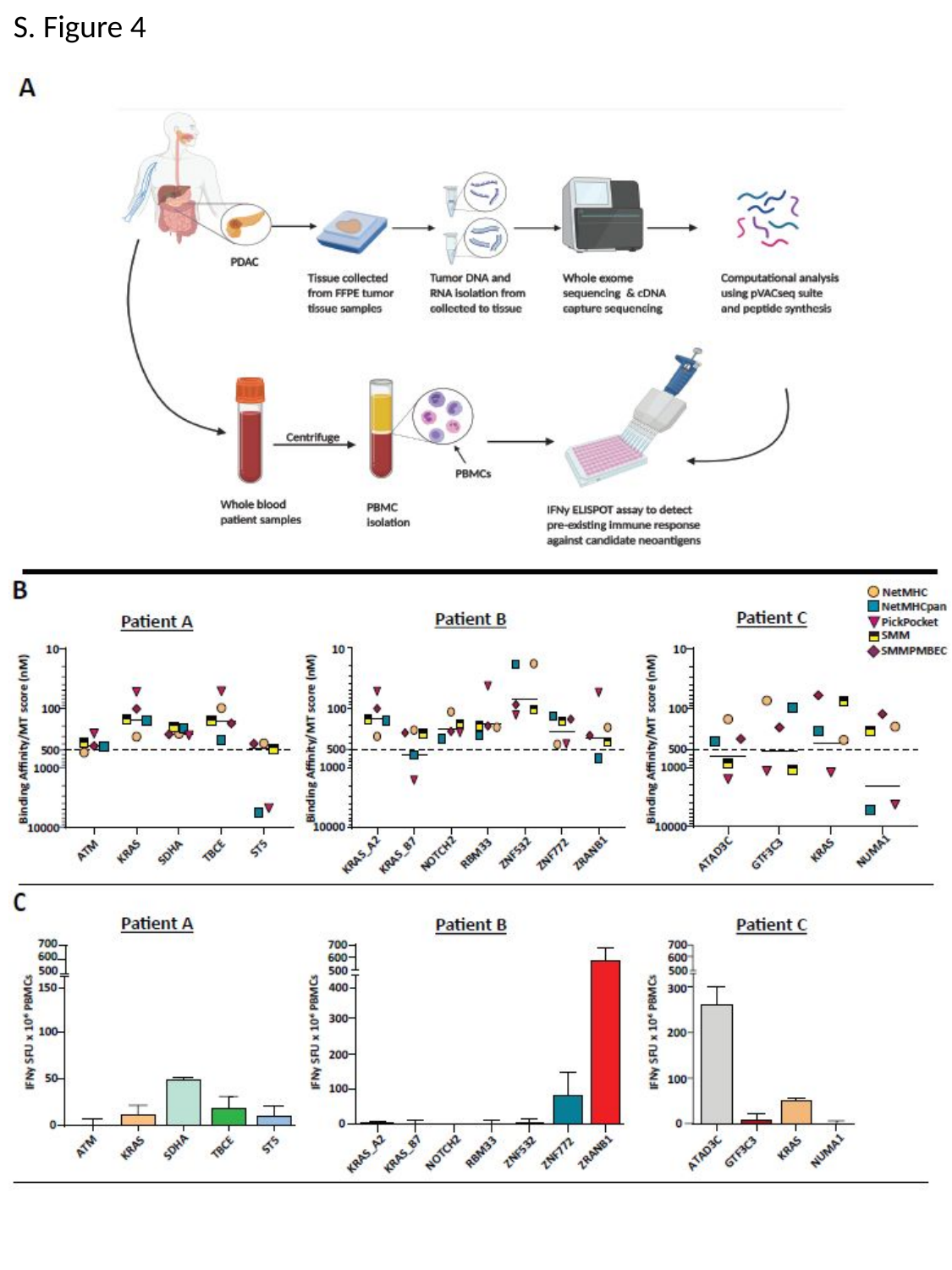

S. Figure 4

## Slide 6
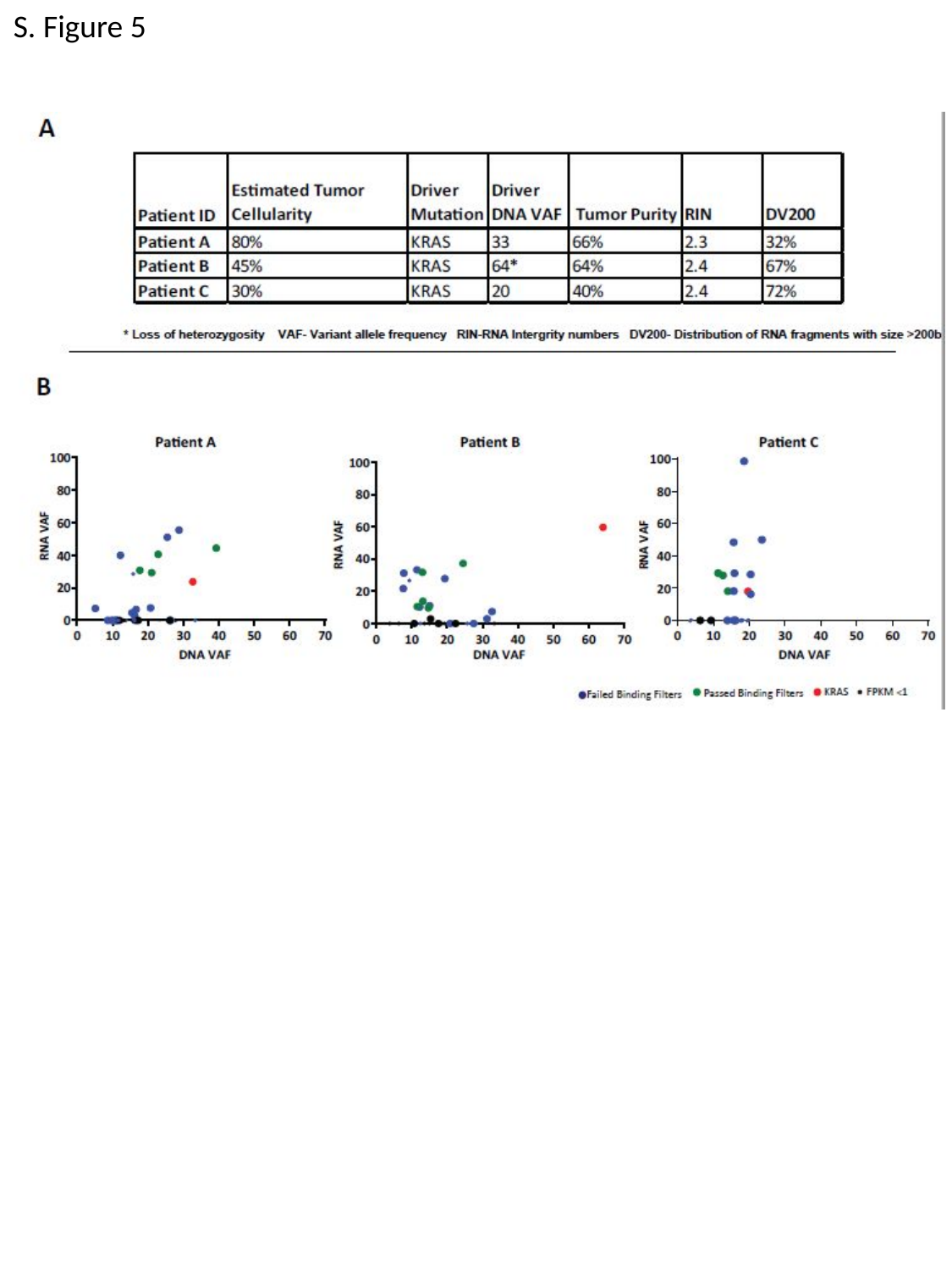

S. Figure 5

## Slide 7
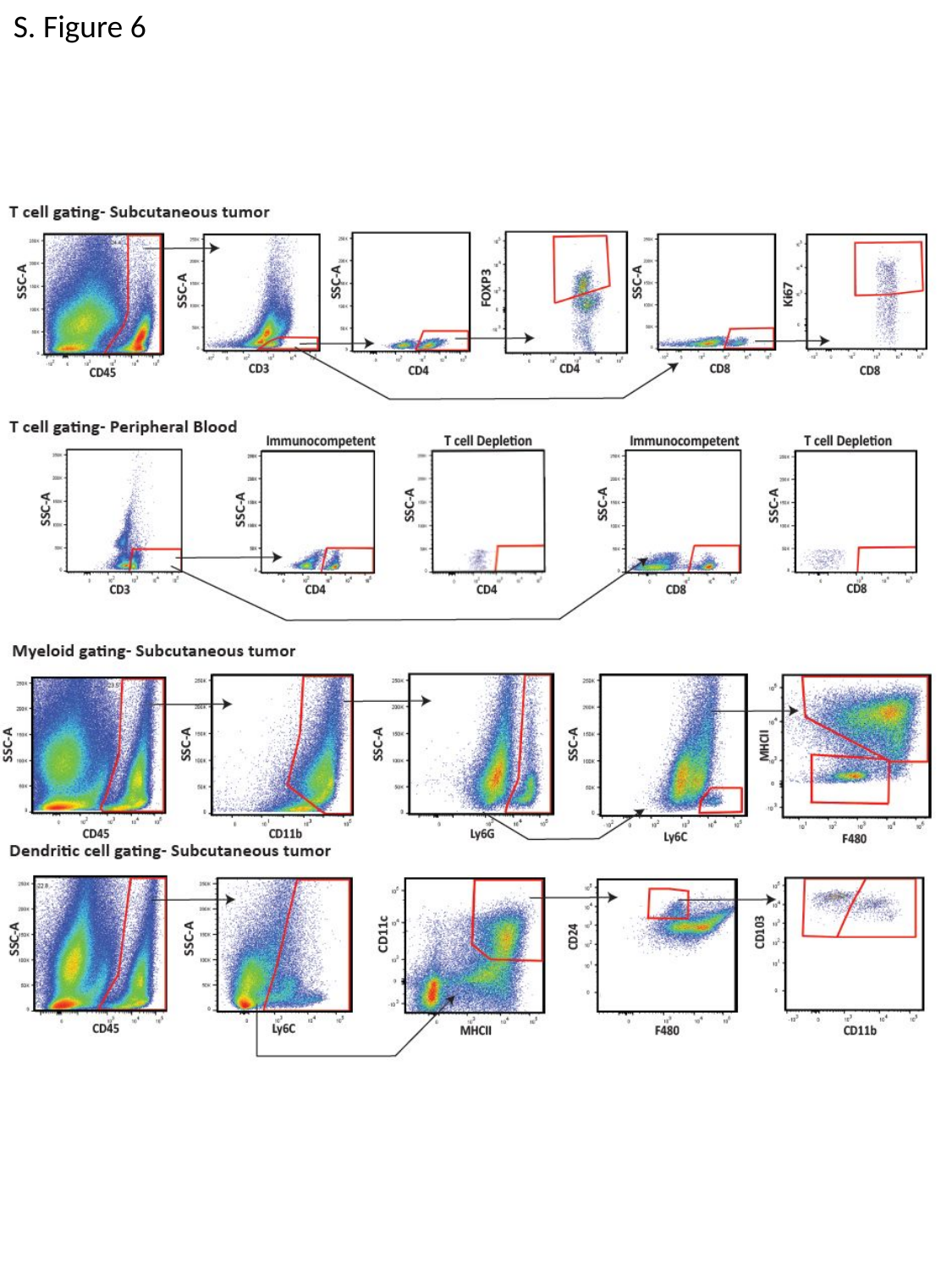

S. Figure 6
