# Supplementary material for: Induction of cancer neoantigens facilitates development of clinically relevant models for the study of pancreatic cancer immunobiology
Source: Cancer Immunol Immunother. 2023 May 13;72(8):2813–27. doi: 10.1007/s00262-023-03463-x (PMC10361914; doi:10.1007/s00262-023-03463-x)
Supplement: Supplementary file 2 — Supplementary file2 (DOCX 22KB) [file 262_2023_3463_MOESM2_ESM.docx]

**Supplementary Table 1:** Predicted neoantigens based on RNA-seq. Treatment with oxaliplatin and olaparib successfully generated high confidence candidate neoantigens compared to the parental KP2 cell line. Amino acid changes between wild type and mutant candidate neoantigens are indicated in red font and underlined. Binding scores <500 nM are considered indicative of strong-intermediate binding. VAF = variant allele frequency.

**Supplementary Figure 1:** A) Heatmap of expression level for genes involved in antigen presentation and processing among clones A, B, and E, and parental KP2 gene expression. B) RNA-seq demonstrates enrichment in genes involved in T cell maturation, cytokine signaling, inflammation, and checkpoint inhibition among clones A and E compared to clone B.

**Supplementary Figure 2:** A) Comparative representation of total CD45+ immune cell infiltration in KP2-OXPARPi clone tumors compared to untreated KP2 tumors. B) Representative IHC images (2X and 10X) IHC images showing the quantification of CD4+ T cell population in KP2 and KP2-OXPARPi tumors. Graphs showing the number of CD4+T cells per cm^2^ of tumor (n=4 to 6 /group). C) Graphs depicting CD4+ and CD8+ T cell frequency in blood, as % of CD45+ cells, of tumor bearing mice treated with vehicle or anti-CD4 and anti-CD8 depletion antibodies.

**Supplementary Figure 3**: Clone D is sensitive to immune checkpoint inhibition while clones C and F are resistant. A) Mice were inoculated with 5*10^5^ tumor cells. Once tumors were palpable, mice were randomized into either treatment with normal saline (NS), or aPD1/aCTLA4 (8-10 mice/group). Tumor volumes were calculated using formula V=1/2(l*w^2^). *=p<0.05, **=p<0.01. B) Flow cytometry performed on single cell suspension of tumor cells of clone F after treatment with aPD1/aCTLA4 or NS. Treatment with aPD1/aCTLA4 do not significantly alter the T-cell infiltrate of clone F tumors.

**Supplementary Figure 4:** Human pancreatic ductal adenocarcinoma expresses neoantigens that can induce T cell responses measured by ELISpot assay. A) Schematic to provide an overview of human neoantigen prediction and immune response detection. Human PDAC tissue were formalin-fixed, and paraffin embedded (FFPE) upon collection. Whole blood samples collected at the time surgery were stored at -80C prior to PBMCs isolation. Punch biopsies (5-6 punches per tissue) were taken from areas with high estimated cellularity. RNA and DNA were purified and sent for exome and cDNA-capture sequencing. To detect the pre-existing neoantigen specific immune response, Peptides synthesized for each identified neoantigen were co-cultured with PBMCs for 48 hours in ELISPOT plates pre-coated with Human IFN-γ. B) Graphs show a summary of binding affinities for each neoantigen predicted to bind with high affinity to the patient’s HLA Class I alleles. Mutations with a binding affinity of < 500 nM on any algorithm were candidate neoantigens (the dashed line indicates a binding affinity of 500 nM). C) Graphs representative of the immune response to the candidate peptides for each patient. Identification of pre-existing immune responses to candidate neoantigens utilizing IFN-γ ELISpot assay. The patient’s PBMCs were cultured with mutant or control peptides for 48 hours. SFU = spot forming unit.

**Supplementary Figure 5:** A) Supplementary Table 1 showing estimated tumor cellularity, DNA VAF, tumor purity, RIN, and DV200 in human PDAC samples. B) Graphs comparing the DNA and RNA VAFs of KRAS driver mutation and other nonsynonymous mutations across human PDAC samples. VAF- Variant allele frequency. RIN- RNA integrity numbers. DV200- Distribution of RNA fragments with size<200bp. FPKM-Fragments Per Kilobase of transcript per Million mapped reads

**Supplementary Figure 6:** Representative flow cytometry gating strategies for myeloid, T cell, and cDC identification. Data are from subcutaneous KP2-OXPARPi tumors and blood samples from tumor bearing mice.
